# Thyroid Hormone Receptor Alpha Is Required for Thyroid Hormone-Dependent Neural Cell Proliferation During Tadpole Metamorphosis

**DOI:** 10.3389/fendo.2019.00396

**Published:** 2019-06-28

**Authors:** Luan Wen, Cara He, Christopher J. Sifuentes, Robert J. Denver

**Affiliations:** Department of Molecular, Cellular and Developmental Biology, The University of Michigan, Ann Arbor, MI, United States

**Keywords:** thyroid hormone (T3), metamorphosis, *Xenopus*, neurogenesis, development, knockout (KO)

## Abstract

Thyroid hormone (T_3_) plays several key roles in development of the nervous system in vertebrates, controlling diverse processes such as neurogenesis, cell migration, apoptosis, differentiation, and maturation. In anuran amphibians, the hormone exerts its actions on the tadpole brain during metamorphosis, a developmental period dependent on T_3_. Thyroid hormone regulates gene transcription by binding to two nuclear receptors, TRα and TRβ. Our previous findings using pharmacological and other approaches supported that TRα plays a pivotal role in mediating T_3_ actions on neural cell proliferation in *Xenopus* tadpole brain. Here we used *Xenopus tropicalis (X. tropicalis)* tadpoles with an inactivating mutation in the gene that encodes TRα to investigate roles for TRα in mitosis and gene regulation in tadpole brain. Gross morphological analysis showed that mutant tadpoles had proportionally smaller brains, corrected for body size, compared with wildtype, both during prometamorphosis and at the completion of metamorphosis. This was reflected in a large reduction in phosphorylated histone 3 (pH3; a mitosis marker) immunoreactive (ir) nuclei in prometamorphic tadpole brain, when T_3_-dependent cell proliferation is maximal. Treatment of wild type premetamorphic tadpoles with T_3_ for 48 h induced gross morphological changes in the brain, and strongly increased pH3-ir, but had no effect in mutant tadpoles. Thyroid hormone induction of the direct TR target genes *thrb, klf9*, and *thibz* was dysregulated in mutant tadpoles. Analysis of gene expression by RNA sequencing in the brain of premetamorphic tadpoles treated with or without T_3_ for 16 h showed that the TRα accounts for 95% of the gene regulation responses to T_3_.

## Introduction

Thyroid hormone (T_3_) plays several key roles in neurological development of vertebrates, influencing dendrite and axon development, synaptogenesis, myelination, cell migration, proliferation, and differentiation (1–3). Thyroid hormone deficiency during fetal and early postnatal life leads to severe, irreversible neurodevelopmental defects in mammals, a condition in humans known as cretinism (1, 4).

The hormone is also essential for development in non-mammalian vertebrates, which is perhaps best exemplified by the dependence of amphibian tadpole metamorphosis on T_3_ (5). Tadpoles of *Xenopus* species are important model organisms for investigating the molecular and cellular mechanisms of T_3_ action during vertebrate development (6).

Thyroid hormone regulates gene transcription by binding to ligand-activated transcription factors (T_3_ receptors; TRs) (7, 8). All jawed vertebrates that have been studied have two TR genes, designated alpha and beta. In mammals, multiple TR isoforms originate from the two genes, some that bind T_3_ and are functional, others that do not bind T_3_. They are produced by differential promoter usage or mRNA processing (3); similar TR protein diversity has not been described in amphibians. Mammalian and amphibian TR genes exhibit cell type and developmental stage-specific expression patterns, which supports that the subtypes/isoforms serve different functions in development and physiology (7, 9, 10). The mRNAs for the *Thra* gene, which codes for the functional isoform TRα1, is widely distributed in rodent brain from early development through adulthood, and most studies support that this protein is the major TR subtype involved with brain development (7). By contrast, mRNAs for *Thrb* (coding for TRβ1 and TRβ2, among several other isoforms) are expressed in the brain during postnatal life, and have a more limited distribution in the brain, being restricted to the retina, cochlea and hypothalamus, and also the pituitary gland (11–13).

The expression patterns of TR genes in *Xenopus* species have broad similarities to mammals. For example, *thra* mRNA is detected in the tadpole shortly after hatching and continues to be produced at a high, mostly constant level through metamorphosis in all tissues that have been studied (5). By contrast, *thrb* mRNA is not detected until the beginning of metamorphosis, at which time it is autoinduced by the rising plasma T_3_ titer. In tadpole brain, *thra* mRNA shows wide distribution; it is detected in virtually all cells in tadpole brain, and exhibits particularly strong signal in neurogenic zones (10, 14). The *thrb* mRNA was detected by *in situ* hybridization histochemistry (ISHH), and the protein by immunohistochemistry (IHC) only after treatment with T_3_, and analysis of TRβ in tadpole brain showed that the protein is found in specific brain nuclei throughout the brain, predominantly outside of neurogenic zones lining the ventricles (10).

Several lines of evidence in mammals support that TRα is critical for mediating T_3_ action on neurogenesis (15), oligodendrocyte differentiation (16), and astrocyte maturation (17). Previously, we showed that the TRα selective agonist CO23 induced brain cell proliferation, while the TRβ-selective agonists GC1 and GC24, used at concentrations that preferentially activate TRβ, had no effect (10). In the current study we used gene knockout technology to investigate a role for TRα in mediating T_3_ action on cell proliferation and gene regulation in tadpole brain. Mitosis is low in the premetamorphic tadpole brain, then it increases strongly and peaks during prometamorphosis, and this depends on endogenous T_3_ (10). We compared the volume and gross morphology of the tadpole brain, and we analyzed mitosis by IHC for phosphorylated histone 3 (pH3) which is a marker for M phase of the cell cycle. We conducted RNA-sequencing (RNA-seq) on a micro-dissected region of the tadpole brain that contains the preoptic area/thalamus/hypothalamus, and which houses neurosecretory neurons that project axons to the median eminence to control pituitary hormone secretion; this tadpole brain region is highly dependent on T_3_ for its development (10, 18, 19). We also conducted time course analyses of gene regulation responses to T_3_ in wild type and mutant tadpole brain.

## Materials and Methods

### Animal Care and Treatment

We obtained wildtype (WT) *Xenopus tropicalis* frogs from NASCO and reared them in dechlorinated tap water at 25C under a 13L:11D photoperiod, and fed them frog brittle (NASCO, Fort Atkinson, WI) or Sera Micron plankton food (tadpoles only). We obtained the heterozygous *thra* mutant frog line (TRαM5) from Dr. Yun-Bo Shi. This line was created using TALENS targeting exon 4 (the third coding exon) of the *X. tropicalis thra* gene, leading to the generation of a mutation that results in a predicted non-sense protein after amino acid 54, which is just before the first zinc finger of the DNA binding domain that begins at amino acid 60 (20). Here we designate the homozygous mutant frog line *thr*a^*mt*−*exon4*^ to indicate the presence of an inactivating mutation in exon 4, leading to a functional knockout.

We generated homozygous *thr*a^*mt*−*exon4*^ tadpoles by crossing heterozygous mutant frogs. Tadpoles were staged using the developmental staging system of Nieuwkoop and Faber (21) (NF). We used PCR-based genotyping to identify homozygous *thr*a^*mt*−*exon4*^ tadpoles as previously described (22). We tail clipped tadpoles at NF stage 48, and lysed the tissues in 30 μL Quick™Extraction buffer at 65°C for 15 min, followed by heating to 94°C for 5 min. To analyze the wild type *thra* allele we used forward primer F_wt_ 5′- AGCTATCTGGACAAAGACGAGCCG-3′, and for the mutant allele we used forward primer F_m5_ 5′-ACATCCCCAGCTATCCCCAGCTATG-3′. The common reverse primer was 5′-GCAAACTTTTTGGCTCAGAGGCCAC-3′. We used the same PCR program for both primer sets: 94°C 30 s, 68°C 60 s for 35 cycles.

For treatment of tadpoles with 3,5,3′-triiodothyronine (T_3_; sodium salt, Sigma-Aldrich, St. Louis, MO), we dissolved the hormone in 0.01N NaOH to make a 500 μM stock solution, then we added the stock to the aquarium water to a final concentration of 5 nM T_3_ for all experiments; controls received an equivalent amount of 0.01N NaOH. For the RNA-seq experiment, we treated NF stage 54 tadpoles with vehicle or T_3_ added to the aquarium water for 16 h before harvesting tissues for RNA isolation. For time course analyses using RTqPCR we treated NF stage 54 tadpoles with T_3_ for 0, 8, 16, or 42 h before harvesting tissues for analysis. For morphometric analyses and immunohistochemistry for cell proliferation we treated tadpoles with T_3_ or vehicle for 42 h before tissue harvest. All procedures involving animals were conducted under an approved animal use protocol (PRO00006809) in accordance with the guidelines of the Institutional Animal Care and Use Committee at the University of Michigan.

For morphometric analyses we euthanized tadpoles or newly metamorphosed frogs by immersion in 0.1% benzocaine, then recorded body weight and body length (using calipers; snout-tail tip for tadpoles, snout-vent for frogs) before dissecting the head and fixing it in 4% paraformaldehyde dissolved in MOPS buffer (0.1 M MOPS, 10 mM EGTA, 1 mM MgSO_4_, pH 7.4; Sigma-Aldrich) at 4°C overnight. We then dissected the fixed tadpole brain and captured images using a Leica stereoscope. All images were captured at the same magnification (10 X) and illumination settings. We used ImageJ software to quantify brain length, width and height for untreated NF stage 56 and 66 animals (*n* = 6 or 8/genotype, NF 56 or 66, respectively). We calculated brain volume by multiplying brain length, width and height, and normalized each animal's brain volume to its body length. We also analyzed brain morphology of NF stage 54 tadpoles after treatment with 5 nM T_3_ for 48 h; here we measured brain length and width (*n* = 5/treatment and genotype).

### Total RNA Extraction, RNA Sequencing, and Reverse Transcriptase Quantitative PCR (RTqPCR)

We micro-dissected the region of the tadpole brain containing the preoptic area/thalamus/hypothalamus for isolation of RNA as previously described (23). For each biological replicate we pooled tissue from 5 tadpoles. We isolated total RNA from tadpole brain using Tri Reagent (Sigma-Aldrich, St. Louis, MO) and the Direct-zol RNA kit (Zymogen) following the manufacturers' instructions. The quality of the RNA was analyzed using a Bioanalyzer, and libraries were prepared by the University of Michigan DNA Sequencing Core. We used 3 biological replicates for each genotype and hormone treatment, for a total of 12 samples sequenced on two lanes using the Illumina Hiseq-4000 platform with 50 nt read length single-end sequencing. We conducted quality processing on raw reads using FastQC (v0.11.7), and we mapped aligned reads to the *X. tropicalis* genome (v4.1) using Bowtie. We conducted gene-level read counts using RSEM to count the number of reads overlapping each of 22,820 custom-annotated *X. tropicalis* genes. This custom annotation (the MNHN gene model) was built by Nicolas Buisine and Laurent Sachs based on their unpublished high-throughput RNA paired-end tag sequencing to identify the 5′ and 3′ ends of transcripts that were expressed in different *X. tropicalis* tissues, including the brain. We then conducted differential expression analysis using limma.

We used RTqPCR to validate gene expression changes identified by RNA-seq, and also for analysis of the kinetics of gene transcription responses to exogenous T_3_ in the two genotypes. We synthesized cDNA from 1 μg total RNA using the High Capacity cDNA Synthesis Kit (Applied Biosystems Inc., Foster City, CA). We conducted real-time qPCR using ABsolute Blue qPCR SYBR Low ROX Mix (ABgene Thermo Scientific, Surrey, UK) and Fast 7500 Real-Time PCR System (ABI) or StepOne Real Time PCR Systems (Life Technologies). We designed oligonucleotide primers to span exon-exon boundaries using the program Prime Blast (NCBI) (Table 1). For most genes we used a relative quantification method (24, 25) to compare mRNA levels by generating standard curves for each gene using a pool of cDNA. To compare levels of *thra* and *thrb* mRNAs in brains of tadpoles of the two genotypes we used an absolute quantification method. We constructed standard curves using the plasmids pCR-TOPO-xtTRα and T7TS-xtTRβ that contain full-length cDNAs for *thra* and *thrb*, respectively. The two cDNA inserts were of similar size (*thra*-1936 nt; *thrb*-2223 nt). We normalized mRNA levels to the reference gene *ef1*α, which did not change after treatment with T_3_ (data not shown).

**Table 1:** Oligonucleotide primer sequences used for reverse transcriptase quantitative polymerase chain reaction.

**Gene name**	DNA sequence*
*cga* F	GCATGTGCTCCATTCCTTTCC
*cga* R	GGCCTTTCCGAGCCTTTTTG
*cbx7* F	TGCCATTGGAGAGCAAGTGT
*cbx7* R	TGCTGTATTTGGGAGGCCAG
*crebrf* F	TGGGCTTTGAGATGCCTCAG
*crebrf* R	TTGCTAGGAGGTCTGTGCTC
*dio3* F	CGCGGCTTGGATGTCATTGC
*dio3* R	GCTCCAGTGACACGCACCTT
*ef1a1* F	CGGAACTACCCTGCTGGAAG
*ef1a1* R	GGCAAAGGTAACCACCATGC
*egln3* F	GACCGACTGGTGATGCTCTG
*egln3* R	CGTTGGATTGTCCACATGGC
*igf2* F	GTTTGTGGAGACAGGGGCTT
*igf2* R	CAGCTCCGGAAGCAACATTC
*klf9* F	GGCACAGGTGTCCTTATGCT
*klf9* R	AAGGGCGTTCACCTGTATGG
*nr3c2* F	GTCCACTATTTCGAGCCCAG
*nr3c2* R	GGCGTTACTCCAGGAAGGAAT
*pim3* F	CGGTGTACACGGATTTTGACG
*pim3* R	ACGGTTGCTGATCTTCCATGA
*st3* F	GGTTATGTGTGGCGCCTTCG
*st3* R	AATGGGAAAGGGCCCAGAGG
*thibz* F	CCAAGGGAAACGGGTGGCTT
*thibz* R	GTGCCACCTCTGCGGAAAGT
*thra* F	AAATGCATTGCCGTTGGCAT
*thra* R	GCCGCTCTCGATTCTCTTCA
*thrb* F	TAGTTAATGCGCCTGAGGGTG
*thrb* R	TGCTCGGAGGGACATGATCT
*traf3* F	GTTCGACTAGCCCACAATGC
*traf3* R	TCCCTCCTGCAGCAGTTATC
*tshb* F	TGTGCTTACTGCCTTGCCAT
*tshb* R	CAGCCAGGAATGGTCACTGT
*znf395* F	CATGTACAAGTGCCTGTGGC
*znf395* R	CTCTCTTGCGCTGGTCAGAA

* *F, forward; R, reverse. Sequences given are 5′ to 3′*.

### Immunohistochemistry (IHC)

We conducted IHC on tadpole brain to detect mitotic cells using a rabbit antiserum raised against human pH3 (phosphorylated histone 3, Millipore), which marks cells in M phase of the cell cycle (10). We analyzed untreated tadpole brains from the two genotypes (*n* = 4–7/genotype) at NF stage 56 when cell proliferation is highest during metamorphosis (10). We also treated NF stage 54 tadpoles of the two genotypes with T_3_ for 42 h (*n* = 4–5/genotype and treatment), which induces a robust cell proliferation response in WT tadpole brain (10). We euthanized animals by immersion in 0.1% benzocaine, then dissected the head and fixed it in 4% paraformaldehyde in MOPS buffer at 4°C overnight. After fixation we dissected the brain from the skull, saturated the tissue in 30% sucrose overnight at 4°C, embedded it in OCT compound (Fisher Scientific), snap-froze the sample, and stored at −80°C until processing. We produced transverse, 14 μm cryosections, dried them for 2 h at 42°C, and then stored the slides at −80°C until analysis. For IHC, we first washed the cryo-sections in Tris-buffered saline plus 0.1% tween-20 (TBST) three times, then blocked with 10% normal goat serum diluted in TBST for 1 h at room temperature. We incubated the sections with primary antibody (1:500 dilution) overnight at 4°C, and then washed with TBST three times. We then detected primary immune complexes by incubating with a secondary antibody conjugated with Cy3 (1:1,000 dilution, Jackson ImmunoResearch Laboratories, Inc., West Grove, PA) at room temperature for 2 h, washed three times in TBST, and mounted the sections using a mounting medium that contained DAPI for counterstaining (Vector Laboratories).

We captured digital micrographic images at 100X using an Olympus IX81 inverted microscope (Olympus, Tokyo, Japan) and a Retiga 1300R Fast digital video camera (QI Imaging, Tuscon, AZ). We carefully matched sections for anatomical level following the *Xenopus* brain atlas developed by Tuinhof et al. (26), with modifications by Yao et al. (27). Images were analyzed in a blinded manner. We randomly selected 5 sections per brain within the region bound by the anterior preoptic area and hypothalamus/thalamus using a random number generator, and we manually counted cells immunoreactive for pH3 (pH3-ir) (10).

### Statistics and Data Analysis

We analyzed data using the computer program SYSTAT (v. 13; Systat Software, San Jose, CA). Differences between treatments were analyzed by one-way ANOVA followed by Fisher's least significant difference (Fisher's LSD) *post hoc*-test, by two-way ANOVA, or by Student's independent sample *t*-test (α = 0.05). Derived values were Log_10_-transformed before statistical analysis if the variances were found to be heterogeneous. We used linear regression analysis to analyze time course gene expression data.

## Results

### Brain Size and Cell Proliferation Are Reduced in *thra^*mt*−*exon4*^* Animals

**Figure 1:**
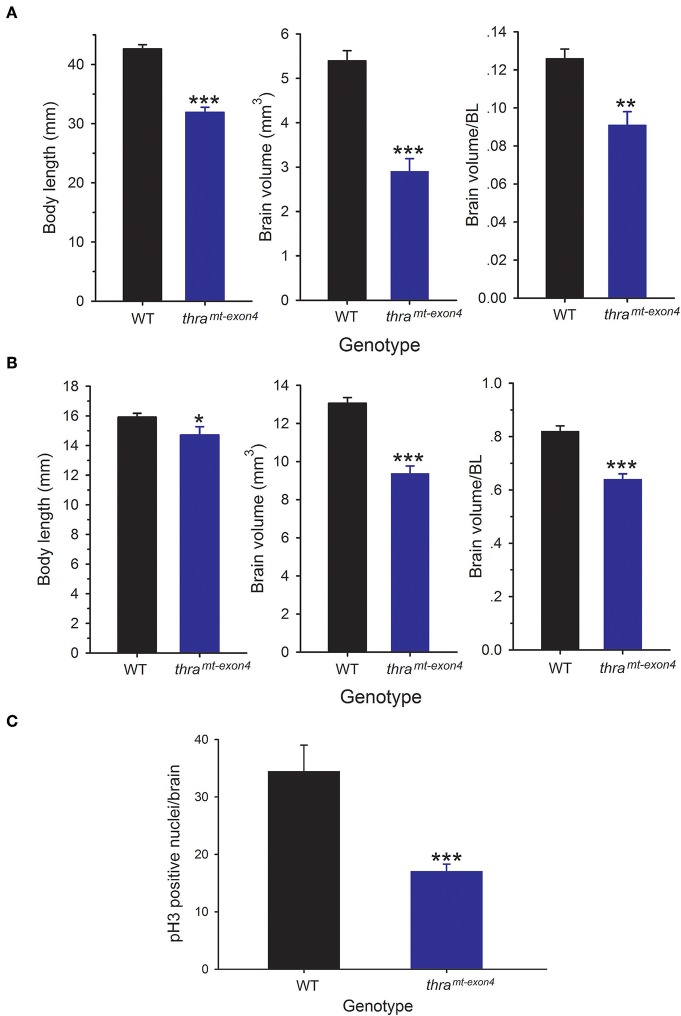
Brain size and cell proliferation is reduced in *thr*a^*mt*−*exon4*^ animals. We measured body length and brain volume in wild type (WT) and *thr*a^*mt*−*exon4*^ animals at two stages of development. Brain volume was measured on fixed brains as described in the Materials and Methods. Bars are the mean ± SEM. **(A)** Comparison of body length and brain volume of WT and *thr*a^*mt*−*exon4*^ prometamorphic tadpoles (NF stage 56; *n* = 6/genotype). **(B)** Comparison of body length and brain volume of WT and *thr*a^*mt*−*exon4*^ newly metamorphosed frogs (NF stage 66; *n* = 8/genotype). **(C)** Comparison of phosphorylated histone 3 (pH3) immunoreactive nuclei in the brain of WT and *thr*a^*mt*−*exon4*^ NF stage 56 tadpoles (*n* = 4–6/genotype). ^*^p < 0.01, ^**^p < 0.001, ^***^p < 0.0001, unpaired Student's *t*-test.

We compared brain size in WT and *thr*a^*mt*−*exon4*^ tadpoles at NF stage 56, the developmental stage when T_3_-dependent neural cell proliferation is maximal (10), and in newly metamorphosed frogs (NF stage 66). The NF stage 56 *thr*a^*mt*−*exon4*^ tadpoles were 25% smaller (body length from snout to tip of tail) than WT animals, but their brain volume was 46% smaller (Figure 1A). Brain volume, corrected for body length was 28% smaller in *thr*a^*mt*−*exon4*^ tadpoles compared with WT. The NF stage 66 (newly metamorphosed frogs) *thr*a^*mt*−*exon4*^ animals were 8% smaller (snout to vent length) than WT animals, but their brain volume was 28% smaller (Figure 1B). Brain volume corrected for body length was 22% smaller in newly metamorphosed *thr*a^*mt*−*exon4*^ frogs compared with WT (Figure 1B).

Our earlier findings showed that cell proliferation in *Xenopus* tadpole brain peaks at NF stage 56 (10). To investigate if the smaller brain size in NF stage 56 *thr*a^*mt*−*exon4*^ tadpoles was correlated with reduced cell proliferation, we conducted immunohistochemistry for pH3, a marker for cells in M phase of the cell cycle. This analysis showed a significant (50.5%) reduction in pH3-immunoreactive cell nuclei in *thr*a^*mt*−*exon4*^ tadpole brains compared with WT (Figure 1C).

### TRα Is Required for T_3_-Dependent Morphological Changes, and Induction of Cell Proliferation in Tadpole Brain

**Figure 2:**
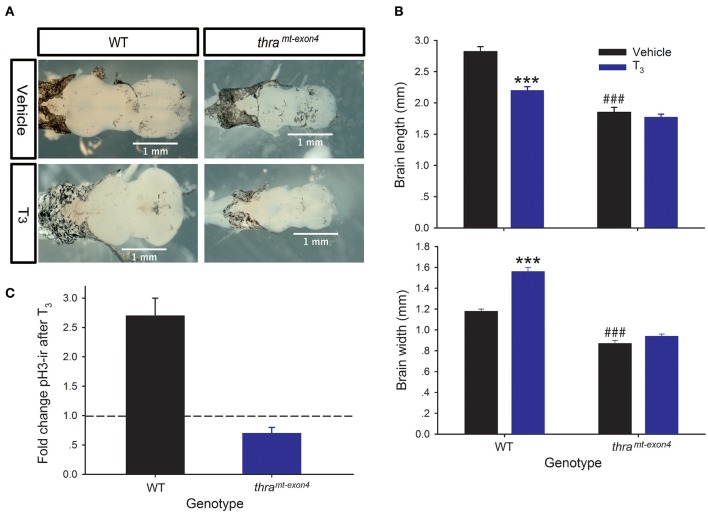
Thyroid hormone receptor α is required for T_3_-dependent morphological changes, and induction of cell proliferation in tadpole brain. We treated wild type (WT) and *thr*a^*mt*−*exon4*^ premetamorphic (NF stage 54) tadpoles with T_3_ (5 nM) or vehicle added to the aquarium water for 42 h, then measured brain size and phosphorylated histone 3 immunoreactivity (pH3-ir) in the brain as described in the Materials and Methods. Bars are the mean ± SEM. **(A)** Exogenous T_3_ caused dramatic morphological changes in the brains of WT, but not *thr*a^*mt*−*exon4*^ tadpoles. Shown are dorsal images of fixed tadpole brains. **(B)** Exogenous T_3_ decreased length, and increased width of WT tadpole brain, but had no effect in *thr*a^*mt*−*exon4*^ animals (^***^p < 0.0001, unpaired Student's *t*-test). In vehicle-treated tadpoles, the baseline brain length and width were both smaller in *thr*a^*mt*−*exon4*^ compared with WT (^*###*^*p* < 0.0001, unpaired Student's *t*-test). **(C)** Treatment with T_3_ strongly increased pH3-ir in WT (*p* < 0.0001, unpaired Student's *t*-test), but decreased it in *thr*a^*mt*−*exon4*^ tadpole brain (*p* < 0.0001). Shown is the fold change in total brain pH3-ir nuclei in each genotype. The dashed line indicates fold change of 1.0 = no change.

Treatment of NF stage 54 (early prometamorphic) WT tadpoles with T_3_ (5 nM for 42 h) caused significant changes in tadpole brain morphology, decreasing brain length by 28%, and increasing brain width by 24% (Figures 2A,B). By contrast, exogenous T_3_ had no significant effect on brain morphology in *thr*a^*mt*−*exon4*^ tadpoles. Our previous work showed that exogenous T_3_ can induce cell proliferation in NF stage 54 *Xenopus* tadpole brain, measured by BrdU incorporation or pH3-ir. We saw the expected increase in pH3-ir after T_3_ treatment in WT tadpoles (~2.7 fold increase), but in *thr*a^*mt*−*exon4*^ tadpoles T_3_ treatment reduced pH3-ir by ~30% compared with vehicle-treated animals (Figure 2C).

### Thyroid Hormone Receptor α Is the Major TR Expressed in Tadpole Brain

**Figure 3:**
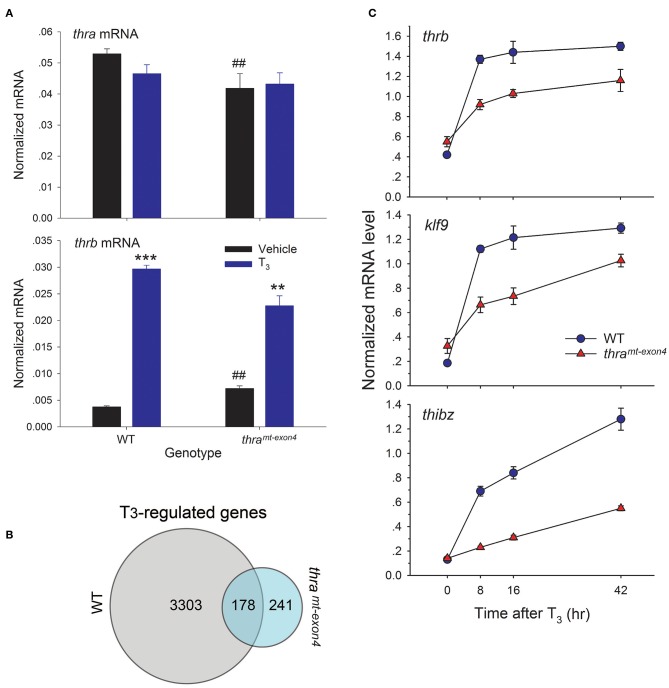
Gene transcription responses to exogenous T_3_ are impaired in *thr*a^*mt*−*exon4*^ tadpole brain. We treated wild type (WT) and *thr*a^*mt*−*exon4*^ premetamorphic (NF stage 54) tadpoles with T_3_ (5 nM) or vehicle added to the aquarium water for different times, then measured target gene mRNA levels in brain (region of the preoptic area/thalamus/hypothalamus) by RTqPCR using absolute quantification (for *thra* and *thrb*, panel A) or relative quantification (genes in panel B). **(A)** Thyroid hormone receptor α is the major TR subtype expressed in tadpole brain. Comparison of baseline mRNA levels, and effects of T_3_ treatment for 42 h on *thra* and *thrb* mRNA levels in WT and *thr*a^*mt*−*exon4*^ tadpole brain. Bars represent the mean ± SEM (*n* = 4/genotype/treatment). Note that the baseline (vehicle-treated) *thra* mRNA level is 14 times greater than *thrb* mRNA in WT tadpole brain. The baseline *thra* mRNA was significantly lower, while the *thrb* mRNA was significantly higher in *thr*a^*mt*−*exon4*^ compared with WT tadpole brain (^##^*p* < 0.001, Student's unpaired *t*-test). Treatment with T_3_ had no effect on *thra* mRNA, but strongly induced *thrb* mRNA in both genotypes (^**^p < 0.001, ^***^p < 0.0001, Student's unpaired *t*-test). **(B)** Venn diagram representing the numbers of T_3_-regulated genes determined by RNA-seq conducted on RNA isolated from brains of wild type and *thr*a^*mt*−*exon4*^ NF stage 54 tadpoles treated with or without T_3_ for 16 h. **(C)** Time course of induction by T_3_ in tadpole brain of mRNAs for three direct T_3_ response genes, *thrb, klf9*, and *thibz* (note that these three genes are included in the 178 genes that overlap between WT and *thr*a^*mt*−*exon4*^). Points represent the mean ± SEM (*n* = 4/genotype/time point). The mRNA level for each of the genes was elevated at 8 h in both genotypes (*p* < 0.001, ANOVA), and the slope of each curve was significantly different between WT and *thr*a^*mt*−*exon4*^ (*p* < 0.0001, linear regression analysis).

Using RTqPCR with absolute quantification, we found that baseline *thra* mRNA was ~12.5 times higher than baseline *thrb* mRNA, confirming that TRα is the major TR subtype in tadpole brain (Figure 3A). The baseline *thra* mRNA was significantly lower in *thr*a^*mt*−*exon4*^ tadpole brain compared with WT (21% lower), while the baseline *thrb* mRNA was significantly higher in *thr*a^*mt*−*exon4*^ (~2 fold; Figure 3A). Treatment with T_3_ for 42 h had no significant effect on *thra* mRNA level in either genotype; whereas, T_3_ treatment caused a large induction of *thrb* mRNA in the brains of WT (7.9 fold increase) tadpoles, and a significant but lower induction in *thr*a^*mt*−*exon4*^ animals (3.2 fold increase).

### The Vast Majority of Gene Regulation Responses to Exogenous T_3_ Are Lost in *thra^*mt*−*exon4*^* Tadpole Brain

We used RNA sequencing to analyze T_3_-dependent changes in gene transcription in tadpole brain after exposure to exogenous T_3_ for 16 h, comparing WT and *thr*a^*mt*−*exon4*^ (NF stage 54). We chose this time point for analysis because it should capture most or all direct T_3_-dependent gene regulation responses, including the delayed immediate early genes (23, 28, 29). We identified 3,481 unique mRNAs that were induced or repressed in WT vs. 419 in *thr*a^*mt*−*exon4*^ (*thr*a^*mt*−*exon4*^ only 12% of WT), and 178 genes that overlapped between the two genotypes (Figure 3B). That is, only 5.1% (178/3481) of genes regulated by T_3_ in WT were regulated in the brain of *thr*a^*mt*−*exon4*^ animals. Interestingly, 241 genes (57.5% of all T_3_ regulated genes in *thr*a^*mt*−*exon4*^) were regulated by T_3_ only in *thr*a^*mt*−*exon4*^ animals. The RNA-seq dataset has been deposited in the Gene Expression Omnibus archive at the National Center for Biotechnology Information (GEO accession #GSE130816).

We investigated the kinetics of transcriptional activation by T_3_ of three well-known direct T_3_ response genes, *thrb, klf9*, and *thibz*. These genes are included in the 178 genes that overlap between WT and *thr*a^*mt*−*exon4*^. All three genes were strongly induced at the 8 h time point in WT tadpole brain, and their mRNAs remained elevated (i.e., they reached a maximum by 8 h: *thrb* and *klf9*) or continued to increase (*thibz*) through 42 h (Figure 3C). By contrast, although all three genes were induced by T_3_ in the brains of *thr*a^*mt*−*exon4*^ tadpoles, the level of induction at 8 h was significantly lower than in WT for all three genes. The kinetics of gene induction, measured as the normalized mRNA level at four time points, and analyzed by linear regression, showed significantly slower kinetics for all three genes in *thr*a^*mt*−*exon4*^ animals (slopes of curves 0 vs. 8 h; *thrb*: WT = 0.95, *thr*a^*mt*−*exon4*^ = 0.37; *klf9*: WT = 0.94, *thr*a^*mt*−*exon4*^ = 0.34; *thibz*: WT = 0.56, *thr*a^*mt*−*exon4*^ = 0.09).

**Figure 4:**
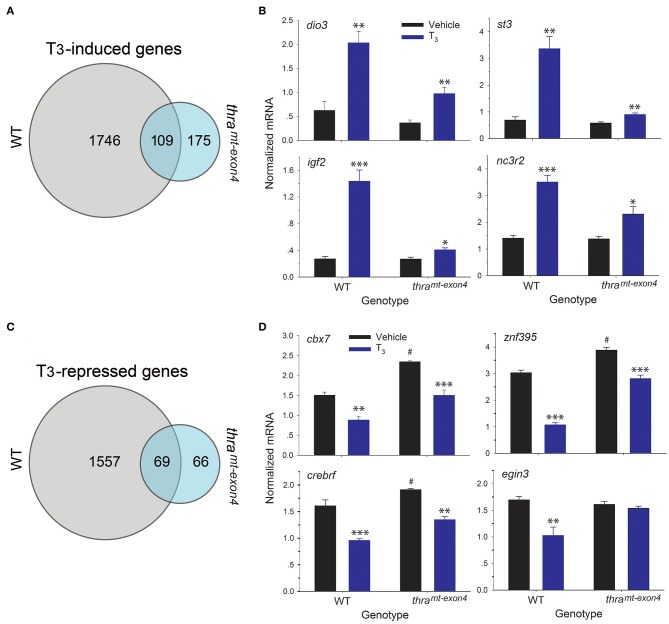
RNA sequencing shows that the majority of gene regulation responses to exogenous T_3_ are lost in *thr*a^*mt*−*exon4*^ tadpole brain. We treated wild type (WT) and *thr*a^*mt*−*exon4*^ premetamorphic (NF stage 54) tadpoles with T_3_ (5 nM) added to the aquarium water for 16 h, then analyzed mRNA levels in brain (region of the preoptic area/thalamus/hypothalamus) by RNA-sequencing or RTqPCR using relative quantification. **(A)** Venn diagram representing the numbers of T_3_-induced genes determined by RNA-sequencing conducted on RNA isolated from tadpole brains treated with or without T_3_. **(B)** Validation of gene regulation for four T_3_-induced genes found within the overlap between the WT and *thr*a^*mt*−*exon4*^ gene sets (109 genes). **(C)** Venn diagram representing the numbers of T_3_-repressed genes determined by RNA-sequencing conducted on RNA isolated from tadpole brains treated with or without T_3_. **(D)** Validation of gene regulation for four T_3_-repressed genes found within the overlap between the WT and *thr*a^*mt*−*exon4*^ gene sets (69 genes). Bars represent the mean ± SEM (*n* = 4/genotype/treatment). For comparisons of vehicle with T_3_ treated: ^*^p < 0.01, ^**^p < 0.001, ^***^p < 0.0001, Student's unpaired *t*-test. For comparisons of baseline mRNA levels (vehicle treated) among the two genotypes: ^#^*p* < 0.01, Student's unpaired *t*-test.

We also compared the induced and repressed gene lists between wild type and *thr*a^*mt*−*exon4*^ tadpoles. This showed that only 5.9% (109 of 1,855) of the genes induced by T_3_ in WT were regulated after loss of *thra* (Figure 4A). Many of the well-known T_3_-induced genes are in this gene list (Supplemental Table 1), suggesting that TRβ is sufficient to mediate T_3_ regulation of a core set of TH response genes. However, in comparing the level of gene induction by T_3_ of these 109 genes (log_2_ fold change >0.5), we found that the majority of these genes exhibited impaired responses to the hormone (Supplemental Table 1); four examples that we validated by RTqPCR are shown in Figure 4B. While exogenous T_3_ caused significant increases in mRNA levels in both genotypes, the level of gene induction was reduced in *thr*a^*mt*−*exon4*^ compared with WT (fold change; *dio3*: WT = 3.2, *thr*a^*mt*−*exon4*^ = 2.6; *st3*: WT = 4.9, *thr*a^*mt*−*exon4*^ = 1.5; *igf2*: WT = 5.3, *thr*a^*mt*−*exon4*^ = 1.5; *nc3r2*: WT = 2.5, *thr*a^*mt*−*exon4*^ = 1.7). There were 169 genes that were induced by T_3_ in *thr*a^*mt*−*exon4*^ but not in WT.

For T_3_-repressed genes, only 4.2% (69 of 1,626) of the genes repressed by T_3_ in WT were regulated after loss of *thra* (Figure 4C). For genes common to WT and *thr*a^*mt*−*exon4*^, the majority had elevated baseline mRNA levels (fold change; *dio3*: WT = 3.2, *thr*a^*mt*−*exon4*^ = 2.6; *st3*: WT = 4.9, *thr*a^*mt*−*exon4*^ = 1.5; *igf2*: WT = 5.3, *thr*a^*mt*−*exon4*^ = 1.5; *nc3r2*: WT = 2.5, *thr*a^*mt*−*exon4*^ = 1.7). In comparing the level of gene repression by T_3_ of these 70 genes (log_2_ fold change > −0.5), we found that many of these genes exhibited reduced responses to the hormone (Supplemental Table 1); three examples that we validated by RTqPCR are shown in Figure 4D. While exogenous T_3_ caused significant decreases in mRNA levels in both genotypes, the level of gene repression was slightly reduced in *thr*a^*mt*−*exon4*^ compared with WT (% change relative to baseline; *cbx7*: WT = −70.2%, *thr*a^*mt*−*exon4*^ = −56%; *znf395*: WT = −184%, *thr*a^*mt*−*exon4*^ = −38%; *crebrf* : WT = −68%, *thr*a^*mt*−*exon4*^ = −42%). Also shown in Figure 4D is one example of a gene (*egin3*) that was repressed by T_3_ in WT (% change relative to baseline: −65%), but was not regulated in *thr*a^*mt*−*exon4*^; also, this gene's baseline mRNA level was not different among the two genotypes. There were 50 genes that were repressed by T_3_ in *thr*a^*mt*−*exon4*^ but not in WT.

Lastly, we identified 10 core cell cycle control genes in the list of genes regulated by T_3_ in WT, and then looked for these genes in the T_3_ regulated list from *thr*a^*mt*−*exon4*^ tadpoles. This showed several cyclins and cyclin-dependent kinases and an E2F transcription factor were regulated by T_3_ in WT brain (Table 2). However, none of these genes were changed by T_3_ in *thr*a^*mt*−*exon4*^ tadpole brain. We will provide a full gene ontology and pathway analysis of T_3_-regulated genes in wild type tadpole brain in a future publication.

**Table 2:** Core cell cycle control genes regulated by T_3_ in wild type by not in *thr*a^*mt*−*exon4*^ premetamorphic tadpole brain^*^.

**Gene name**	**Gene symbol**	**Induced (I) or repressed (R)**
Cyclin J	ccnj	I
Cyclin D2	ccnd2	I
Cyclin-dependent kinase 2	cdk2	I
Cyclin-dependent kinase 8	cdk8	I
Cyclin-dependent kinase 11b	cdk11b	I
Cyclin-dependent kinase 13	cdk13	I
E2F transcription factor 6	e2f6	I
Cyclin-dependent kinase 19	cdk19	R
Cyclin I	ccni	R
Cyclin G2	ccng2	R

* *Data from RNA sequencing conducted on wild type and thra^mt−exon4^ NF stage 54 tadpole brain (diencephalon) treated with or without T_3_ (5 nM) for 16 h*.

**Figure 5:**
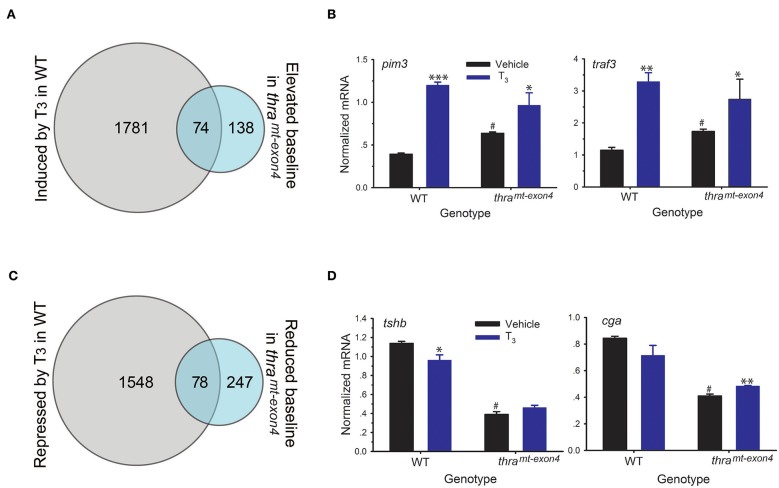
Effects of loss of TRα on baseline gene transcription in premetamorphic tadpole brain. We treated wild type (WT) and *thr*a^*mt*−*exon4*^ premetamorphic (NF stage 54) tadpoles with T_3_ (5 nM) added to the aquarium water for 16 h, then analyzed mRNA levels in brain (region of the preoptic area/thalamus/hypothalamus; including the pituitary) by RNA-sequencing or RTqPCR using relative quantification. **(A)** Venn diagram representing the number of T_3_-induced genes in WT and the number of genes whose baseline mRNA level was elevated in *thr*a^*mt*−*exon4*^ tadpoles, determined by RNA-sequencing conducted on RNA isolated from tadpole brains treated with or without T_3_. **(B)** Validation of gene regulation for two T_3_-induced genes found within the overlap between the T_3_ induced WT and elevated baseline in *thr*a^*mt*−*exon4*^ gene sets (74 genes). **(C)** Venn diagram representing the number of T_3_-repressed genes in WT and the number of genes whose baseline mRNA level was reduced in *thr*a^*mt*−*exon4*^ tadpoles, determined by RNA-sequencing conducted on RNA isolated from tadpole brains treated with or without T_3_. **(D)** Validation of gene regulation for two T_3_-repressed genes found within the overlap between the T_3_ repressed WT and reduced baseline in *thr*a^*mt*−*exon4*^ gene sets (78 genes). Bars represent the mean ± SEM (*n* = 4/genotype/treatment). For comparisons of vehicle with T_3_ treated: ^*^p < 0.01, ^**^p < 0.001, ^***^p < 0.0001, Student's unpaired *t*-test. For comparisons of baseline mRNA levels (vehicle treated) among the two genotypes: ^#^*p* < 0.01, Student's unpaired *t*-test.

Thyroid hormone receptors, in the absence of ligand, are resident in chromatin bound to DNA, where they generate a closed chromatin state and repress gene transcription by recruiting corepressor complexes (8). Unliganded TRs function to repress gene transcription in premetamorphic tadpoles prior to endogenous TH synthesis, and this action is hypothesized to be important for maintaining the tadpole stage, a life cycle stage important for growth and dispersal (i.e., the dual-function model) (30). We hypothesized that loss of TRα would lead to de-repression of genes that are typically induced by T_3_, which would be reflected in increased baseline mRNA levels for these genes in *thr*a^*mt*−*exon4*^ tadpoles. Our RNA-seq analysis found 212 genes whose baseline mRNA levels were increased in *thr*a^*mt*−*exon4*^ tadpoles compared to WT; however, only 74 of these genes (35%) were regulated by T_3_ in WT (Figure 5A). Two examples of these genes analyzed by RTqPCR are shown in Figure 5B (fold change in baseline in *thr*a^*mt*−*exon4*^, *pim3*: 1.6; *traf3*: 1.5; see also *thrb* in Figure 3A). Of the 212 genes, 138 exhibited increased baseline mRNA levels *thr*a^*mt*−*exon4*^, but these genes were not regulated by T_3_ in WT.

We also found 325 genes whose baseline mRNA level was reduced in *thr*a^*mt*−*exon4*^ tadpoles compared with WT (Figure 5C). Of the 325 genes, 78 (25%) corresponded to genes that were repressed by T_3_ in WT. The remaining 247 genes with elevated baseline mRNA levels in *thr*a^*mt*−*exon4*^ were not regulated by T_3_ in WT. Two examples of these genes analyzed by RTqPCR are shown in Figure 5D, one that was repressed by T_3_ in WT (*tshb*: change in baseline in *thr*a^*mt*−*exon4*^ = −66%) and one that was not significantly affected by T_3_ in WT (*cga*: change in baseline in *thr*a^*mt*−*exon4*^ = −51%). These two genes code for subunits of the glycoprotein hormone thyroid stimulating hormone (TSH; *tshb*—TSHβ, *cga*—common glycoprotein subunit alpha); note that the region of the tadpole brain that we dissected for RNA-seq analysis contained the pituitary gland.

## Discussion

Here we show that TRα is required for T_3_-dependent cell expansion in *Xenopus* tadpole brain during metamorphosis, which supports previous findings that used non-genetic approaches (10). The *thra* mRNA is 135 times more abundant than *thrb* mRNA, supporting that this is the major TR subtype in premetamorphic tadpole brain (10, 31). Furthermore, we found that TRα is required for 95% of the gene regulation responses to T_3_ in premetamorphic tadpole brain. Genes that were regulated by T_3_ in both genotypes exhibited impaired gene regulation responses in TRa knockout tadpole brain, both in their kinetics and magnitude of induction.

In the unliganded state, TRs are resident in chromatin where they recruit co-repressors to generate a compact chromatin state, and repress gene transcription. Hormone binding to TRs results in the exchange of co-repressors for co-activators, which generates an open chromatin environment promoting gene transcription (8). Current evidence supports that TRs have two general roles in tadpoles related to their repressor and activator functions, which is referred to as the dual function model (30, 32–34). During premetamorphosis, before the thyroid gland is developed and producing T_3_, when existing TRs are in the unliganded form, TRs (predominantly TRα) repress gene transcription required for transformation of the tadpole into the juvenile adult, thereby maintaining the tadpole stage. When T_3_ production rises during prometamorphosis, the aporeceptor is converted to a transcriptional activator, recruiting histone modifying enzymes that generate an open chromatin structure required for active transcription. The TRα is hypothesized to be necessary to establish competence of cells to respond to T_3_ (30).

Earlier findings from our laboratory showed that mitosis in neurogenic zones of the tadpole brain increased dramatically at the beginning of metamorphosis, and reached a peak at NF stage 56, which correlated with rising plasma T_3_ concentration (10). Neural cell proliferation can be induced precociously by the addition of T_3_ to the aquarium water of premetamorphic tadpoles (NF stage 50–52); whereas, we found that cell proliferation in prometamorphic tadpole brain was reduced to premetamorphic levels by treatment with the goitrogen methimazole, which blocks the endogenous rise in plasma T_3_. We also provided strong evidence that the action of T_3_ on neural cell proliferation is mediated by TRα. For example, using ISHH and IHC, we found that TRα is highly expressed in neurogenic zones of tadpole brain, with strongest expression in proliferating cells. Furthermore, treatment with the TRα selective agonist CO23 induced mitosis. By contrast, we found that TRβ was expressed outside of neurogenic zones where neural cells undergo migration and differentiation, and treatment of tadpoles with two TRβ selective ligands, GC1 and GC24, at concentrations that preferentially activate TRβ, failed to induce cell proliferation (10, 35). These findings, using pharmacological agents combined with histochemistry, support the view that the TRα mediates hormone action on mitosis in tadpole brain.

Our current findings using mutant tadpoles deficient in TRα provide additional support for this model. We found that the *thr*a^*mt*−*exon4*^ tadpoles had proportionally smaller brains corrected for body size, both during prometamorphosis when cell proliferation is maximal, and in the newly metamorphosed juvenile frog. This may be explained by our finding that early prometamorphic *thr*a^*mt*−*exon4*^ tadpoles had fewer pH3-positive cell nuclei in their brains compared with WT. These differences between the two genotypes is likely due to complete impairment of T_3_ dependent cell proliferation, since we found that premetamorphic *thr*a^*mt*−*exon4*^, unlike WT tadpoles, were totally resistant to exogenous T_3_ effects on gross morphological changes, and pH3-ir in the brain. Taken together with our previous work (10), these new findings provide strong support for an essential role for TRα in T_3_-dependent neurogenesis in *Xenopus* tadpole brain.

We saw a small (~30%), but statistically significant decrease in pH3-ir after T_3_ treatment in *thr*a^*mt*−*exon4*^ tadpole brain. This may be due to the upregulation of *thrb* in the absence of the proliferative actions of liganded TRα, since several lines of evidence support that TRβ functions to limit cell proliferation, and induce cell differentiation. The level of *thrb* mRNA in tadpole brain is low or non-detectable during premetamorphosis, then rises during prometamorphosis in response to increasing plasma T_3_ titer, and peaks at metamorphic climax when most cells in the brain have exited the cell cycle, migrated, and are in the process of differentiating and maturing (10, 23). Experiments using the TRβ specific T_3_ analog GC1 support the view that TRβ functions in cell differentiation and apoptosis (10, 36). Thyroid hormone receptor β is expressed predominantly outside of neurogenic zones in tadpole brain (10), consistent with a role in cell migration and differentiation. We hypothesize that the reduction in pH3-ir that we observed in *thr*a^*mt*−*exon4*^ tadpole brain after treatment with T_3_ is due to the actions of TRβ, which is autoinduced in *thr*a^*mt*−*exon4*^ tadpole brain as it is in WT (see Figure 3A). With the loss of TRα, the ability of T_3_ to induce cell proliferation is lost; whereas, induction of TRβ may promote neural progenitors to exit the cell cycle and to differentiate.

Our results support that TRα is the major TR subtype expressed in tadpole brain, and its loss leads to profound deficits in gene regulation responses to T_3_. Until now, analysis of gene regulation in *thra* mutant tadpoles was limited to a handful of known T_3_ regulated genes (20, 22, 37, 38). Here we used RNA-seq after treatment with T_3_ to provide an unbiased, transcriptomic analysis of the effects of loss of TRα on gene regulation in tadpole brain. This showed that only 5.1% of the genes regulated by T_3_ in WT tadpole brain were similarly regulated in *thr*a^*mt*−*exon4*^ animals. Furthermore, genes that were regulated by T_3_ in both genotypes exhibited impaired responses to the hormone in *thr*a^*mt*−*exon4*^ tadpole brain. For three well-known direct T_3_ response genes (*thrb, klf9, thibz*), we saw impaired kinetics in response to the hormone, and also a reduction in the maximal level of gene induction. This suggests that, while this subset of genes can be regulated by TRβ, and indeed, their delayed response likely reflects the time required to autoinduce *thrb* and produce functional TRβ protein, TRα is required for the initial and maximal response to the hormone. Taken together, the data support that TRα is required for cells to become competent to respond to the hormone, and to maintain a sustained response (30).

Interestingly, we found that 57.5% of the genes regulated by T_3_ in *thr*a^*mt*−*exon4*^ were only regulated in the mutant genotype and not in WT. This may be explained by TRα having a normal function of counteracting T_3_ actions on gene transcription mediated by TRβ, which are unmasked when TRα is lost. This is supported by findings in the mammalian pituitary thyrotrophic cell line Tα1T, where TRα is recruited to the *Tshb* gene promoter only after knockdown of TRβ (39).

The loss of TRα also affected the baseline mRNA level of some genes (212 increased, 325 decreased in *thr*a^*mt*−*exon4*^). However, only 35% of the genes whose baseline mRNA levels were increased after loss of TRα were induced by T_3_ in WT. The increase in baseline mRNA levels of T_3_ regulated genes after loss of TRα is likely due to loss of the repressor actions of unliganded TRα, as has been shown previously (20, 22, 37, 38). Interestingly, baseline transcription (vehicle treated) of a subset of genes (325) was reduced in the absence of TRα, supporting that this unliganded nuclear receptor is necessary to support gene transcription in the absence of hormone. Of these genes, 24% were also repressed by T_3_ in WT. Taken together, our findings support that TRα is not only required for gene regulation responses to T_3_ in tadpole brain, but also plays an important role in maintaining normal baseline gene transcription. This supports studies conducted on other tadpole tissues that showed dysregulation of baseline gene transcription in TRα knockout tadpoles (20, 22, 37, 38).

Lastly, our RNA-seq analysis provides a molecular basis for the loss of T_3_-dependent cell proliferation that we saw in *thr*a^*mt*−*exon4*^ tadpole brain. We found 10 core cell cycle control genes that were induced by T_3_ in WT, but not in *thr*a^*mt*−*exon4*^ tadpole brain. These included several cyclins, cyclin-dependent kinases and an E2F transcription factor. Taken together, our findings support the view that the TRα is the major TR subtype expressed in tadpole brain, and it is required for T_3_ action on cell proliferation. The vast majority of genes regulated by T_3_ in tadpole brain, both induced and repressed, depend on TRα.

## Data Availability

The datasets generated for this study can be found in GEO, GSE130816.

## Ethics Statement

All procedures involving animals were conducted under an approved animal use protocol (PRO00006809) in accordance with the guidelines of the Institutional Animal Care and Use Committee at the University of Michigan.

## Author Contributions

LW designed and conducted experiments, analyzed data, and helped to write the manuscript. CH conducted experiments, analyzed data, and helped to write the manuscript. CS analyzed data and helped to write the manuscript. RD designed experiments, analyzed data, and wrote the manuscript.

### Conflict of Interest Statement

The authors declare that the research was conducted in the absence of any commercial or financial relationships that could be construed as a potential conflict of interest.
